# Quantifying Protein Function Specificity in the Gene Ontology

**DOI:** 10.4056/sigs.561626

**Published:** 2010-03-30

**Authors:** Brenton Louie, Silas Bergen, Roger Higdon, Eugene Kolker

**Affiliations:** 1 Bioinformatics and High-throughput Analysis Laboratory, Seattle Children’s Research Institute, Seattle, WA, USA; 2 Predictive Analytics, Seattle Children’s Hospital; 3 Department of Biostatistics, University of Washington School of Public Health; 4 Biomedical and Health Informatics Division, Department of Medical Education and Biomedical Informatics, University of Washington School of Medicine, Seattle, WA, USA

**Keywords:** protein annotation, protein function, function specificity

## Abstract

Quantitative or numerical metrics of protein function specificity made possible by the Gene Ontology are useful in that they enable development of distance or similarity measures between protein functions. Here we describe how to calculate four measures of function specificity for GO terms: 1) number of ancestor terms; 2) number of offspring terms; 3) proportion of terms; and 4) Information Content (IC). We discuss the relationship between the metrics and the strengths and weaknesses of each.

## Introduction

Genomic sciences and biological understanding can be greatly enriched by quantitative comparisons between the descriptions of protein functions [1-5]. To achieve this, numerical descriptions of protein function specificity must be defined. This is now possible using the Gene Ontology (GO [6,7], ). The GO is a standardized description of protein function structured as a hierarchy of “parent-child” relationships, formally called a directed acyclic graph (DAG). DAGs have long been used in computer science as a mathematical formalism for describing complex objects. Modeling protein function as a DAG provides a means of more precisely defining protein function and the relationship between functions as opposed to traditional natural language descriptions which are information-rich but unfortunately not amenable to computers.

The use of the GO provides a conception of function specificity that has immediate implications in the automated annotation of proteins [4,6]. Millions of proteins in public databases have their functions inferred from proteins with similar sequences. The meaningful transfer of those functions is made possible in part by the standardized organization of the GO. The GO is organized so that as one traverses away from the root node function, definitions become narrower; examples of broad functional terms are “*catalytic activity*” (GO:0003824) or “*transporter activity*” (GO:0005215), while narrower functions would be “*adenylate cyclase activity*” (GO:0004016) or “*peptidoglycan transporter activity*” (GO:0015647) [6]. Quantifying the path from broad to narrow function specificity is vague however as path lengths are variable and there are no edge weights which makes meaningful numeric interpretation of function specificity problematic. This ambiguity can be addressed by considering various aspects of the DAG structure of GO. Each node in GO (i.e., GO term) is assigned a function and measurements such as the number of ancestor or offspring nodes for that term can be used to give a numeric assessment of that term’s specificity. This paper discusses various methods created to improve the precision of assigning and comparing specificity of GO terms and discusses strengths and weaknesses of each method.

## Requirements

The methods described here utilize the R programming language with the Bioconductor R package installed and a dataset of associations between gene or protein identifiers and GO terms, e.g. the gene2go file available at ftp://ftp.ncbi.nlm.nih.gov/gene/DATA/. All methods described here are available for free.

## Procedure

### Number of Ancestors

**Figure 1:**
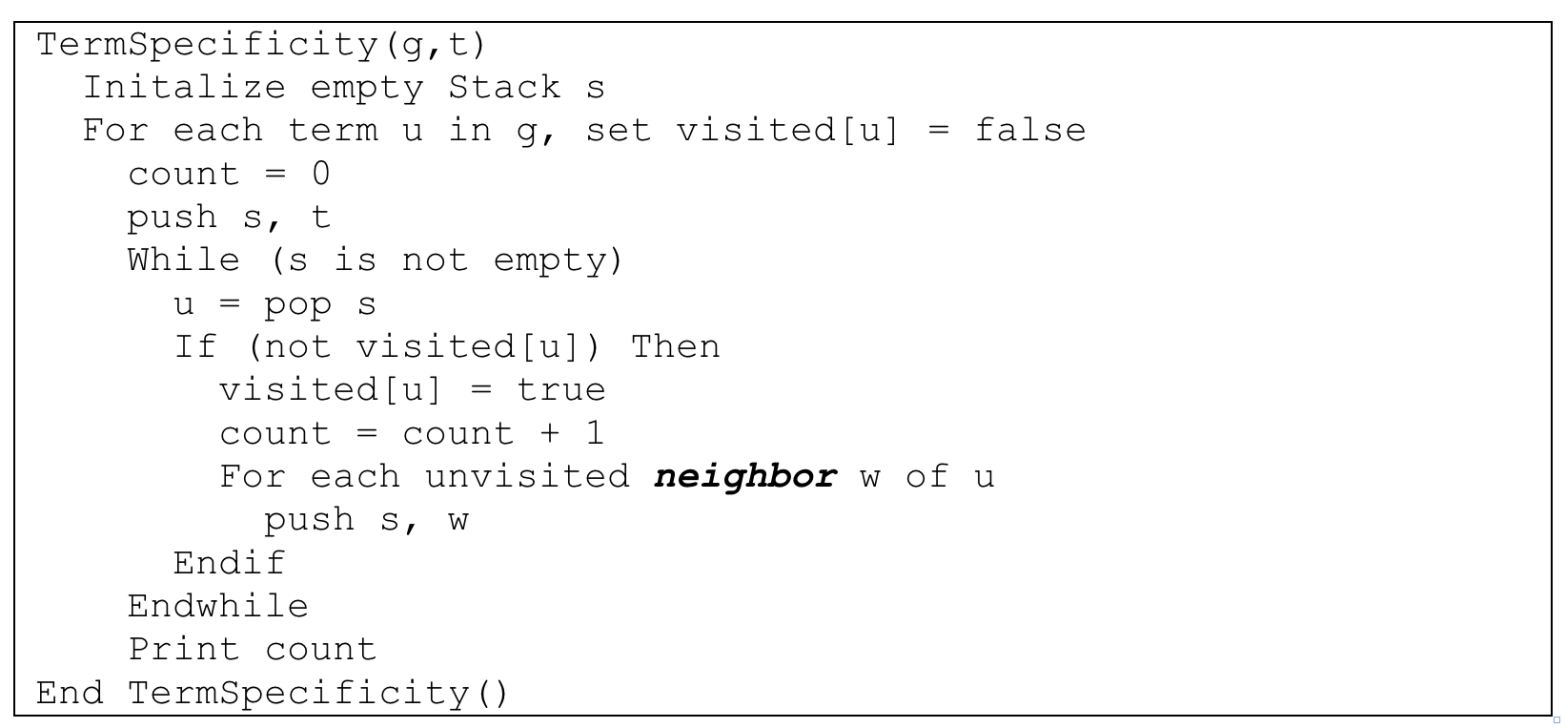
Function specificity in the GO. This graph illustrates, using a simplified model of the GO hierarchy, how the number of ancestors (Ancestors) and offspring (Offspring) for GO terms would be calculated. For calculating IC, the number of proteins with a particular GO term annotation must be accounted for in determining their probability of occurring in the data set (Proteins). The probability of a term occurring depends on whether or not a particular term or any of its offspring terms occurs in a data set (Total Proteins). Details on how each specificity measure is calculated can be found in the “Procedures” section.

**Figure 2:**
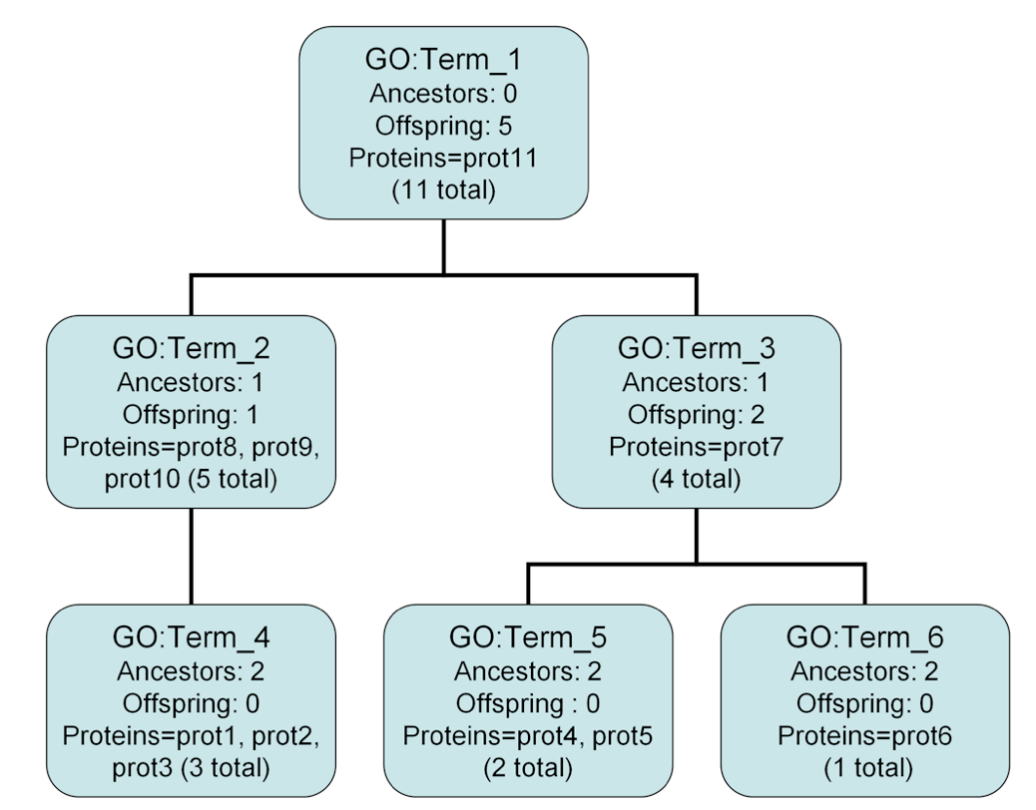
Pseudocode for determining ancestors or offspring in the GO. The “g” and “t” arguments to the TermSpecificity function are the GO structured as a DAG, and the particular term in question respectively. This routine is similar to a standard “depth-first-search” graph algorithm that searches for a specific node in a subgraph [8], however its purpose is to count the visited nodes rather than to locate a specific node. An “unvisited” neighbor is either a parent or child node, depending on whether offspring or ancestors are being counted. For the implementation of these methods see the “Implementation” section.

One of the original ways to describe the function specificity of a given GO term is through the number of ancestor nodes a term has which is a measure of how “deep” a GO term is in the GO hierarchy. This is calculated by counting the number of ancestor terms for a given GO term up to and including the root term; in general one would expect a more specific function to have a greater number of GO ancestors. Refer to Figure. 1 : the GO term GO:Term_1 has no ancestors; the GO terms GO:Term_2 and GO:Term_3 each have one ancestor, etc. This approach appeals to the nature of the GO since as one traverses down the GO away from the root node one expects the specificity of terms to increase. Pseudocode for this process can be seen in Figure 2. Note that in determining ancestors, the “neighbors” of a GO term would only include parent nodes (not child nodes).

### Number of Offspring

A second method of measuring GO term specificity is the number of offspring nodes a GO term has. As with the number of GO ancestors, this is a useful metric in that it takes under immediate consideration the hierarchical nature of the GO with more specific terms tending to have fewer offspring. We created an adjusted, more normalized measurement of GO offspring (Offsp_N_) in order to have the measure increase with specificity in the same manner as GO ancestors by considering the maximum number of offspring (i.e., the number of offspring of the root node, which is 8267 for the version of the GO molecular function ontology concurrent with this paper), and calculated this measure as follows:


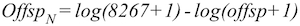


The interpretation of this measure is that higher Offsp_N_ indicates more specific function. This maintains the same “higher is better” idea consistent with other GO specificity metrics such as GO ancestors. Pseudocode for this calculation is also exemplified in Figure 2. Note however that the neighbors of a node would only include its child nodes – not its parents.

### GO Proportion

In order to take under consideration both the number of ancestor nodes and the number of offspring nodes of a particular GO term we created a measure of function specificity that incorporates both measures. This measure is a proportion of the offspring of a term over the total number of “reachable” terms, or offspring + ancestors. In order to have the same interpretation as the above GO ancestor and offspring measures, we took 1 minus the proportion, as follows:


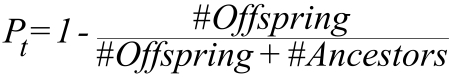


Where *P_t_* is the GO proportion of the reachable terms for term *t*. The 0-1 range of this proportion, with 0 indicating non-specific function and 1 indicating high specificity, is useful for a readily-understood interpretation when comparing terms.

## Information Content

Information content (IC) provides an alternate measure of function specificity. Unlike the previous specificity metrics IC is a function of a data set. In particular, it is related to the probability of the occurrence of a particular GO term in a database of protein annotations. GO terms that occur less frequently have higher IC and are assumed to be more specific. The formula for calculating IC is:


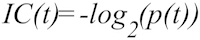


Where *t* is a particular GO term and *p* is the probability of that term occurring in a data set. The probability of a term occurring is the frequency with which that particular term or any of its ancestors occur in a data set [9,10]. For any GO term in the ontology, *p(t)* is calculated as follows:

Step 1: Count the number of proteins assigned to the term.

Step 2: Count the number of proteins assigned to all offspring of the term.

Step 3: Add the counts from Steps 1 and 2 and divide by the total number of proteins in the data set.

Consider, for example, Figure 2. To calculate *p(t)* and IC for GO:Term_3, we follow these steps:

Step 1: Count the proteins assigned to GO:Term_3 (one).

Step 2: Count the proteins assigned to the offspring of GO:Term_3 (three).

Step 3: Calculate *p(t)* for GO:Term_3, in this case 4/11. Its IC is therefore 1.5.

The appealing part IC is that it implicitly accounts for the hierarchical structure of the GO. The root node, or “*molecular function*” (GO:0003674), has IC of 0.0, creating an appealing baseline measurement of the most non-specific function. As the GO is traversed away from the root node one generally expects IC for terms to increase.

## Implementation

### Calculating GO Ancestors/Offspring

The GO.db package from the Bioconductor library written for the R statistical software provides a quick and easy way of obtaining the ancestor and offspring terms of any given GO term [11]. The library contains the GO DAG and outputs descriptions of GO term relationships with a few simple commands. The Bioconductor library can be obtained free of charge from the following website: http://www.bioconductor.org/docs/install/. After this library and the GO.db package have been installed, these commands are what would be used to obtain all the ancestor GO terms of, for example, the GO term “*collagen binding*” (GO:0005518):

> GOMFANCESTOR[[“GO:0005518”]]

 [1] “all” “GO:0005515” “GO:0003674” “GO:0005488”

This returns the ancestors of the term GO:0005518, which in this case are “*binding*” (GO:0005488), “*protein binding*” (GO:0005515), and the root term “*molecular function*” (GO:0003674). The *length* function in R can be used to count the terms in the returned vector from the GOMFANCESTOR function. Analogously, to obtain the offspring of a specific term, one would use the command

> GOMFOFFSPRING[[“GO:0005518”]]

 [1] “GO:0070052”

Which returns the lone offspring of GO:0005518, which is the term “*collagen V binding*” (GO:0070052).

### Calculating Information Content

**Figure 3:**
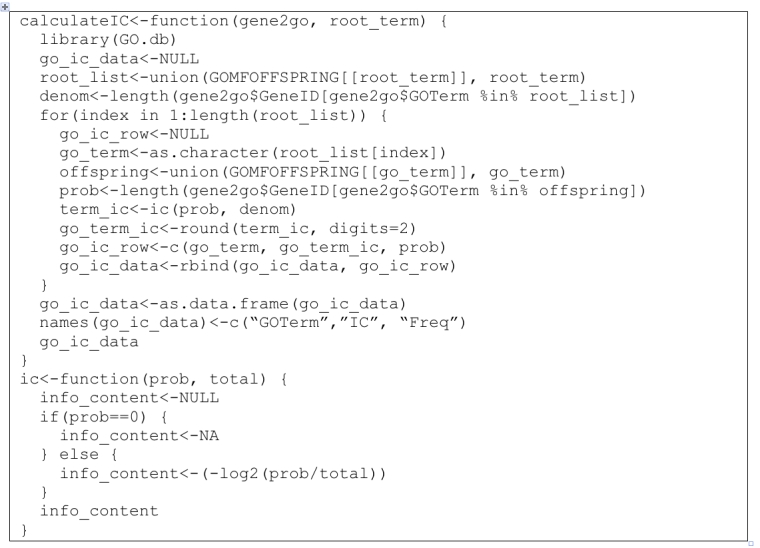
Calculation of IC, implemented in the R programming language. The “root_term” is “GO:0003674” for the root term in the GO molecular function hierarchy. The “gene2go” argument is the gene2go file available at ftp://ftp.ncbi.nlm.nih.gov/gene/DATA, or an equivalent file with associations between genes/proteins (a “GeneID” column header) and GO terms (“GOTerm” column header).

As discussed above, IC is related to the probability of a specific GO term occurring in a data set. The below R code that calculates IC takes as input the “gene2go” file which is a data set that contains association of proteins with GO terms (the gene2go file can be found at ftp://ftp.ncbi.nlm.nih.gov/gene/DATA/); and the “root_term” which is “GO:0003674” for the molecular function ontology. Code, implemented in the R programming language, can be seen in Figure 3.

## Discussion

**Figure 4:**
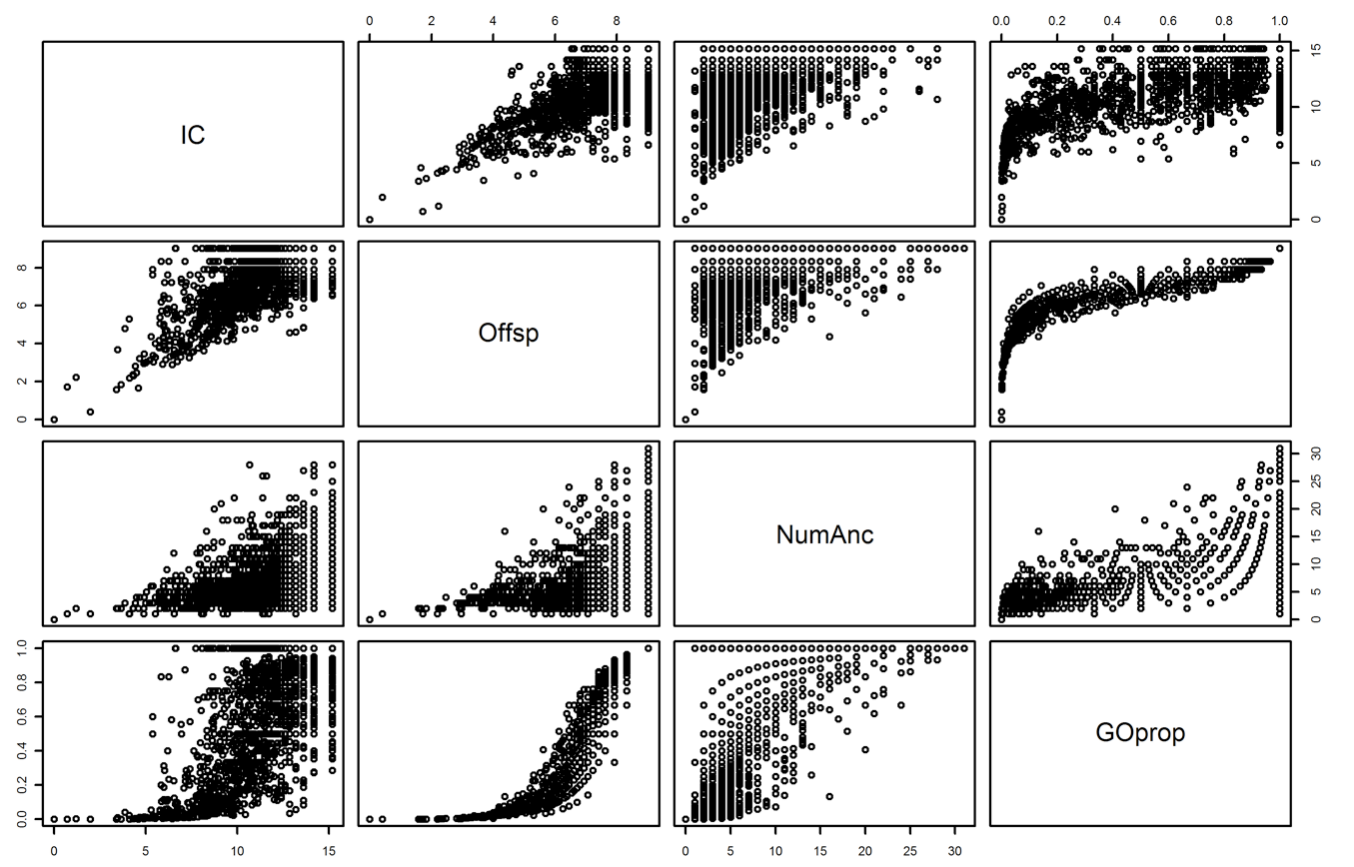
Correlation between different measurements of function specificity. This scatter-plot matrix of each pair of specificity measures shows the ways in which they are correlated with each other. Each individual row and column contains comparisons for the particular variables with the rows corresponding to the y-axis and columns to the x-axis. For instance, for the IC and Offsp plot in the lower quadrant, the y-axis is Offsp (0-8) and the x-axis is IC (0-15). In the upper quadrant this is reversed. The variables in the pairs plot are the aforementioned IC and Offsp (normalized); as well as NumAnc (the number of GO ancestors of a term); and GOprop (the proportion considering both number of ancestors and number of offspring of a term). As is shown in this graph, there is strong correlation between each of the variables but not exactly, indicating each one has information about specificity another might not have.

The methods described above provide a useful toolkit of varied approaches to measuring function specificity using the Gene Ontology. The goal is to develop a metric of specificity that is comparable among all GO terms, which is not an easy task. Each method has information about the specificity of any particular GO term that another might not have (see Figure 4), and each has their own strengths and weaknesses. The number of GO ancestors of a term as discussed above is useful in that it directly reflects the DAG nature of the GO, is easy to calculate, and has an intuitively appealing interpretation. The problem with this measurement is that there are idiosyncrasies in the GO that can cause the path length between any given GO term and the root node to be highly variable. For instance, the GO term GO:0000102 “*catalysis of the transfer of L-methionine from one side of a membrane to the other, up its concentration gradient*” (GO:0000102) has no offspring and 17 ancestors, while the term “*the action of a molecule that contributes to the structural integrity of a cytoskeletal structure*” (GO:0005200) has no offspring and two ancestors. The variability inherent in the number of ancestors of a term makes it difficult to accurately assess and compare specificity between GO terms, making it the least useful specificity metric in our opinion.

The measurement based on the normalized number of GO offspring also is a function purely of the GO, like that of GO ancestors, but is more reliable due to its lower variability. The GO offspring measurement will also remain consistent across data sets, providing a non-data-dependent option of measuring and comparing function specificity in contrast to IC (see below). Note that GO offspring does in fact correlate fairly strongly with IC (Figure 4). The caveat to the use of this normalized offspring metric as a measure of function specificity is that there are GO terms seemingly quite specific by other metrics still have many offspring as well as some seemingly non-specific terms by other metrics that have few or no offspring (see Table 1).

**Table 1:** Examples of GO terms and their specificity metrics.

	GO Offspring	Offsp_N_	GO Anc	GO Prop	IC
Max Specificity Value	0	9.02	31	1	15.18
Min Specificity Value	8267	0	0	0	0
GO:0005524	0	9.02	8	1	6.64
GO:0003700	1	8.33	5	0.83	5.85
GO:0042302	5	7.23	2	0.29	15.18

Note that GO Offspring is the untransformed number of offspring for a term and Offsp_N_ is the normalized metric. These GO terms provide some examples of how various metrics may provide different “views” regarding the specificity of a term. For instance GO:0005524 is the most specific by Offsp_N_ (9.02) yet is only moderate by IC (6.64), meaning that although it is most specific by the hierarchical structure of the GO, it is a relatively common term in the database. Conversely, GO:0042302 is the most specific by IC (15.18) yet is only moderately specific by Offsp_N_ which means that although more specific functions are possible according to the GO hierarchy, this term is very rare in the database. The GO:0003700 term shows relatively moderate specificity for all metrics.

The GO proportion is useful in that it considers the implications of both the number of ancestors and the number of offspring. It is also on a 0-1 scale, which allows a readily accessible min-max interpretation. However, the concerns with this proportion measure are similar to those pertaining to GO ancestors and GO offspring.

The most unique aspect of IC is that it is calculated from data. The strength of IC, however, doubles as a caveat: since it is data dependent, the IC of a particular GO term may fluctuate from data set to data set. In our experience however the IC calculation is generally robust for most terms, especially common ones as their probability of occurrence in a data set changes little. Very scarce GO terms can be impacted more significantly if more instances of the term are added to a dataset, although we have found this to be a rare event. Also, if a GO term does not occur in a data set then calculating its IC is impossible. This is undesirable, especially if that GO term is of some interest to the research project at hand. Also, occurrence of GO terms in a data set might be due to bias in the way proteins are annotated which may not be representative of the natural state of function specificity, i.e. more specific IC may not be truly indicative of more specific function.

There is also some “divergence” between the various specificity metrics for some GO terms. Concrete examples of where the various specificity measures may diverge can be seen in Table 1. For instance the GO term “*interacting selectively and non-covalently with ATP, adenosine 5'-triphosphate*” (GO:0005524) has the maximum value of the GO offspring measurement (Offsp_N_=9.02) and the GO proportion is 1.0, both indications of very specific function. The IC however is only 6.64, an indication of relatively non-specific function. This exemplifies a case where two of the measures indicate specific function while another indicates moderately-specific function. A reversal of sorts can be seen with the GO term “*the action of a molecule that contributes to the structural integrity of a cuticle*” (GO:0042302). This GO term is very specific by IC (15.18), yet middling specificity is indicated by Offsp_N_ (7.23), and low specificity as indicated by GO proportion (0.29) and GO ancestors (2). The GO term “*the function of binding to a specific DNA sequence in order to modulate transcription*” (GO:0003700), can be seen as a more “middle ground” as all of its metrics indicate relatively moderate specificity. These numbers provide evidence that each of these measurements have information to give about function specificity. Consider that GO offspring, which only takes into account the structure of the GO beneath a particular term, is a product of abstracted biological knowledge as it currently exists. This knowledge appears to be represented to some degree idiosyncratically between different types of functions. In contrast, IC, which only considers the current distribution of GO terms in a database and may be biased due to the experimental methodologies used to annotate proteins. Certainly no metric is perfect, each has strengths and weaknesses, and considering all of them provides a more holistic knowledge of a GO term to enable specificity comparisons across terms.

## References

[1] Kolker E Purvine S Galperin MY Stolyar S Goodlett DR Nesvizhskii AI Keller A Xie T Eng JK Yi E Initial Proteome Analysis of Model Microorganism *Haemophilus influenzae* Strain Rd KW20. J Bacteriol 2003; 185:4593-4602 10.1128/JB.185.15.4593-4602.2003 12867470 PMC165749

[2] Raghunathan A Price ND Galperin MY Makarova KS Purvine S Picone AF Cherny T Xie T Reilly TJ Munson R In Silico Metabolic Model and Protein Expression of *Haemophilus influenzae* Strain Rd KW20 in Rich Medium. OMICS 2004; 8:25-41 10.1089/153623104773547471 15107235

[3] Kolker E Makarova KS Shabalina S Picone AF Purvine S Holzman T Cherny T Armbruster D Munson RS Kolesov G Identification and functional analysis of 'hypothetical' genes expressed in *Haemophilus influenzae*. Nucleic Acids Res 2004; 32:2353-2361 10.1093/nar/gkh555 15121896 PMC419445

[4] Kolker E Picone AF Galperin MY Romine MF Higdon R Makarova KS Kolker N Anderson GA Qiu X Auberry KJ Global profiling of *Shewanella oneidensis* MR-1: Expression of hypothetical genes and improved functional annotations. Proc Natl Acad Sci USA 2005; 102:2099-2104 10.1073/pnas.0409111102 15684069 PMC548307

[5] Louie B Higdon R Kolker E A Statistical Model of Protein Sequence Similarity and Function Similarity Reveals Overly-Specific Function Predictions. PLoS ONE 2009; 4:e7546 10.1371/journal.pone.0007546 19844580 PMC2760442

[6] Ashburner M Ball CA Blake JA Botstein D Butler H Cherry JM Davis AP Dolinski K Dwight SS Eppig JT Gene ontology: tool for the unification of biology. The Gene Ontology Consortium. Nat Genet 2000; 25:25-29 10.1038/75556 10802651 PMC3037419

[7] The Gene Ontology Consortium. The Gene Ontology in 2010: extensions and refinements. Nucleic Acids Res 2010;38:D331-D335 10.1093/nar/gkp1018 19920128 PMC2808930

[8] Chang S. Data structures and algorithms. University of Pittsburg, USA: World Scientific; 2003.

[9] Lord PW Stevens RD Brass A Goble CA Investigating semantic similarity measures across the Gene Ontology: the relationship between sequence and annotation. Bioinformatics 2003; 19:1275-1283 10.1093/bioinformatics/btg153 12835272

[10] Pesquita C Faria D Bastos H Ferreira AE Falcão AO Couto FM Metrics for GO based protein semantic similarity: a systematic evaluation. BMC Bioinformatics 2008; 9:S4-S4 10.1186/1471-2105-9-S5-S4 18460186 PMC2367622

[11] Gentleman RC Carey V Bates D Bolstad B Dettling M Dudoit S Ellis B Gautier L Ge Y Gentry J Bioconductor: open software development for computational biology and bioinformatics. Genome Biol 2004; 5:R80 10.1186/gb-2004-5-10-r80 15461798 PMC545600

